# Mass spectrometric monitoring of interfacial photoelectron transfer and imaging of active crystalline facets of semiconductors

**DOI:** 10.1038/ncomms14524

**Published:** 2017-02-22

**Authors:** Hongying Zhong, Juan Zhang, Xuemei Tang, Wenyang Zhang, Ruowei Jiang, Rui Li, Disong Chen, Peng Wang, Zhiwei Yuan

**Affiliations:** 1 Mass Spectrometry Center for Structural Identification of Biological Molecules and Precision Medicine, Institute of Public Health and Molecular Medicine Analysis, Key Laboratory of Pesticides and Chemical Biology, Ministry of Education, College of Chemistry, Central China Normal University, Wuhan, Hubei 430079, China

## Abstract

Monitoring of interfacial electron transfer (ET) *in situ* is important to understand the ET mechanism and designing efficient photocatalysts. We describe herein a mass spectrometric approach to investigate the ultrafast transfer of photoelectrons that are generated by ultraviolet irradiation on surfaces of semiconductor nanoparticles or crystalline facets. The mass spectrometric approach can not only untargetedly detect various intermediates but also monitor their reactivity through associative or dissociative photoelectron capture dissociation, as well as electron detachment dissociation of adsorbed molecules. Proton-coupled electron transfer and proton-uncoupled electron transfer with radical initiated polymerization or hydroxyl radical abstraction have been unambiguously demonstrated with the mass spectrometric approach. Active crystalline facets of titanium dioxide for photocatalytic degradation of juglone and organochlorine dichlorodiphenyltrichloroethane are visualized with mass spectrometry imaging based on ion scanning and spectral reconstruction. This work provides a new technique for studying photo-electric properties of various materials.

## Abstract (web summary)

Monitoring interfacial electron transfer in photocatalytic systems is fundamentally important but experimentally challenging. Here the authors use mass spectrometry to detect and monitor intermediates formed through photoelectron transfer and to image active crystalline facets of semiconductor photocatalysts.

Photo-induced heterogeneous electron transfer (ET) across the interface between semiconductors and adsorbed molecules has been involved in various photocatalytic reactions^1^,^2^,^3^ and applications to solar energy conversion^4^,^5^,^6^ or environmental cleanup^7^,^8^,^9^. Because of the rapid recombination of photo generated electrons and holes, it still remains challenging to study the mechanisms of ultrafast electron transfer processes with current techniques^10^,^11^,^12^. Ideally, those techniques not only should have the capabilities for charge trapping but also should be able to detect and identify various known/unknown resultant species, short lived intermediates, as well as location of active crystalline facets with high-spatial resolution. Microscopic fluorescence imaging based on organic dye probes has recently been developed as an emerging approach for sensitive detection of resultant reactive oxygen species and the visualization of active facets on individual semiconductor particles^13^,^14^,^15^. In this technique, organic dye probes are usually designed to have two parts: a fluorogenic chromophore for microscopic detection and a reactive site for linking with known or predicted products of photocatalytic reactions. Other unknown or unpredicted species that cannot be recognized by the probe will not be detected. Because of the structural versatility of intermediates and products, this targeted technique cannot provide detailed structural and electronic information on the overall photocatalytic reactions. In addition, to understand the formation mechanisms of intermediates and final products, energies needed for chemical bond cleavages are required but they are not available by using fluorescence imaging techniques alone.

In this work, a mass spectrometric approach was designed to untargetedly detect products along with intermediates of photocatalytic reactions and visualize active crystalline facets. Recently, we have reported a new ionization method based on interfacial photoelectron transfer for mass spectrometric imaging^16^. This work is further aimed at facet-dependent photocatalytic activities of semiconductor crystalline materials, as well as interactions of photoelectrons with adsorbed molecules. Advantages of mass spectrometers for such studies include the high-vacuum sample chamber and the built-in static electric field with laser pulses. The high-vacuum chamber eliminates interferences of abundant atmospheric N_2_ and O_2_, as well as solvents. It also simplifies theoretical calculations that have been used for evaluation of bond energy, ion stability, reaction thermodynamics and kinetics. The built-in electric field facilitates instant separation of photo-generated electron–hole pairs as soon as the laser beam irradiates on surfaces of semiconductors. Once photoelectrons are captured by adsorbed molecules, resultant negatively charged radical anions are pulled out of the surfaces of semiconductor nanoparticles in the static electric field. Therefore various intermediates that are under-revealed previously may be detected with mass spectrometers. In addition, because kinetic energies of photoelectrons are controllable through adjusting the bias voltage between the sample plate and the aperture, it is very convenient to monitor the energies needed for chemical bond cleavage. Compared with other existing spectroscopic methods such as fluorescence or electron spin resonance, the unique feature of mass spectrometry is the capability to provide information on both masses and charges that are essential for structural interpretation of intermediates and final products.

In this work, the proposed mass spectrometric approach has been applied to investigate photocatalytic reactions of juglone and organochlorine 4, 4′-dichlorodiphenyltrichloroethane (DDT) on surfaces of semiconductor nanoparticles. Different intermediates and product ions have been found through associative or dissociative photoelectron capture dissociation, as well as electron detachment dissociation. In addition to well recognized proton-coupled electron transfer, radical initiated polymerization or hydroxyl radical abstraction have also been unambiguously demonstrated with the mass spectrometric approach. In contrast to optical microscopy which shows the physical shapes, mass spectrometric imaging reveals spatial distribution of ions. Active crystalline facets of titanium dioxide have been visualized by scanning all know and unknown degradation products or intermediate ions. It is shown that this mass spectrometric approach should be able to provide a new way for exploring photo-electric properties of various materials.

## Results

### Mass spectrometric monitoring of photoelectron transfer

**Figure 1:**
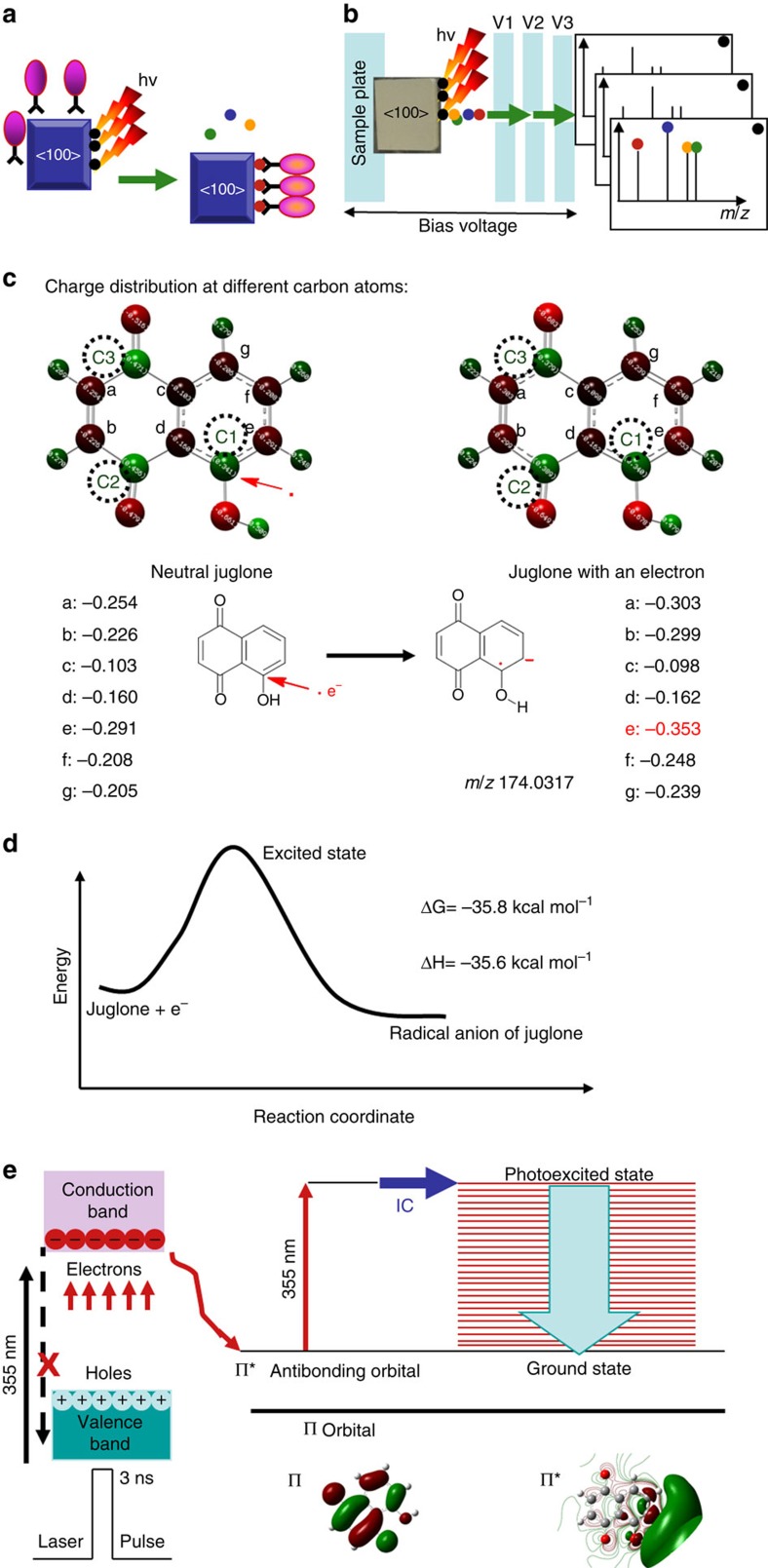
Experimental design and theoretical investigation of interfacial photoelectron transfer. (**a**) Principle of fluorescence spectroscopy-based approach. (**b**) Principle of mass spectrometry-based approach. (**c**) Charge distribution of neutral juglone and the radical anion of juglone. (**d**) Energy profile of photoelectron capture by neutral juglone. (**e**) Formation of anionic ground states through ultrafast internal conversion. Purple ovals: fluorogenic chromophores. Black balls: molecules adsorbed on different sites of semiconductor facets. Coloured balls (red, green, orange and blue balls): resultant intermediates and products of potocatalytic reactions. V1, V2 and V3: the voltages applied to the extraction plate, hexapole and aperture, respectively.

Compared with the general setup of a microscopic fluorescence imaging approach shown in Fig. 1a, by which only predicted species are detected, the mass spectrometry-based approach takes the full scan manner and all ions except radicals and neutral species are detected. As shown in Fig. 1b, an air dried titanium dioxide crystal (rutile) with exposed <100> facet that has been soaked in the solution of electron acceptor juglone was stuck to a conductive alumina tape before the assembly was fixed to the sample plate. In the quadrupole time-of-flight mass spectrometer, ultraviolet laser pulses are synchronized with the detector. While the laser beam (355 nm) scans across the facet <100> point by point, photoelectrons are instantly captured by adsorbed electron acceptor molecules (black balls) and photo-generated ions (coloured balls) are recorded by the detector. Mass spectra are then reconstructed to image site-specific photocatalytic reactions. This approach can also be used for studies of photoelectron transfer from bulk semiconductor nanoparticles. In such case, a suspension of nanoparticles in isopropanol was pipetted onto the sample well. After air-dried, the sample plate was then subjected to ultraviolet irradiation.

Juglone and DDT have been chosen for proof-of-principle demonstration. Because the wavelength of the laser used in this work is 355 nm, we have first checked the ultraviolet absorption spectra of these two compounds in order to ensure that their ionization is not due to direct ultraviolet absorption. Supplementary Figure 1 shows both of them do not have strong absorption at 355 nm. In addition, production of photoelectrons, when light is shone onto a material has been well known. To confirm that the ionization of juglone and DDT surely result from the capture of photoelectrons, insulated glue tapes have been put on the surface of the sample plate to block the interfacial transfer of photoelectrons, as shown in Supplementary Fig. 2. It was found that no signal can be obtained. Without the use of semiconductors, direct irradiation of ultraviolet light on juglone or DDT cannot cause the formation of ions that can be detected by the mass spectrometer.

### Associative trapping and detection of photoelectrons

So far, photoelectrons or holes generated on surfaces of semiconductors have rarely been directly observed with current techniques. Photoelectron spectroscopy is one of the alternatives but it does not provide information on interfacial interactions of photoelectrons with adsorbed molecules^17^,^18^. Although these interactions can be monitored by chemical reactions of fluorescence dye probes with resultant reactive oxygen species or hydroxyl radical^19^,^20^,^21^, experimental evidences are needed for further validation because how and why reactive oxygen species or hydroxyl radicals are produced upon light irradiation remain unknown. The strategy described herein is to use an electron acceptor such as juglone as the molecular probe to trap photoelectrons. In the absence of O_2_, capture of photoelectrons switches neutral juglone molecules to negatively charged radical anions that can be detected in the negative ion mode of the mass spectrometer. Once radical anions are formed, they are instantly pulled out of the surfaces of semiconductors in the static electric field. Detection of radical anions provides direct experimental evidence on the interfacial electron transfer and photoelectron capture. As shown in Fig. 1c, calculations with density functional theory (DFT) indicate that there are three charge deficient carbon atoms labelled as C1, C2 and C3 respectively in the neutral juglone. These carbon atoms have possibilities to capture photoelectrons. With an acquired electron, the carbon atom labelled as e of the juglone becomes the most negatively charged carbon atom which implicates the preferable electron capture at the carbon atom labelled as C1. Actually, it can be found that the acquired negative charges and unpaired electrons present in resultant radical anions are delocalized over the whole ions, when photoelectrons are captured at this position. Delocalization of charges stabilizes the radical anion and promotes the proceeding of such interfacial charge transfer. In contrast, although carbon atoms labelled as C2 and C3 are also charge deficient, acquired charges by these two carbon atoms cannot be as well delocalized as that of the carbon atom labelled as C1. The energy profile of the photoelectron trapping process is shown in Fig. 1d. Changes of free energies and enthalpies after neutral juglone traps low energy photoelectrons are −35.8 and −35.6 kcal mol^−1^, respectively. Therefore photoelectron trapping by juglone is an exothermic process that can spontaneously proceed. It has been theoretically demonstrated that trapping of photoelectrons by juglone molecules is energetically favourable.

As we know, radical anions have been considered as highly reactive species^22^,^23^,^24^. The time for an ion generated in the sample chamber to be detected by a quadrupole time-of-flight mass analyser is usually about in the scale of microsecond. Detection of such intermediate radical anions of juglone implicates an ultrafast relaxation mechanism that can account for the ability of juglone to trap and retain photoelectrons. By using a combination of time-resolved photoelectron spectroscopy and high level *ab initio* calculation, Verlet *et al*. has demonstrated that formation of anionic ground states through ultrafast internal conversion attributes to the stability of radical anions of *para*-benzoquinone, which is a derivative of juglone^25^. As shown in Fig. 1e, internal conversion would re-distribute excess internal energy among all vibrational modes that can be quenched by the surroundings. Because internal conversion process is in the timescale of sub-40 fs, which is much faster than that of detection time (∼μs), it is feasible to observe anionic radicals in stable ground state. So it has been theoretically demonstrated that the detection of intermediate radical anions resulting from the trapping of photoelectrons is also energetically favourable.

**Figure 2:**
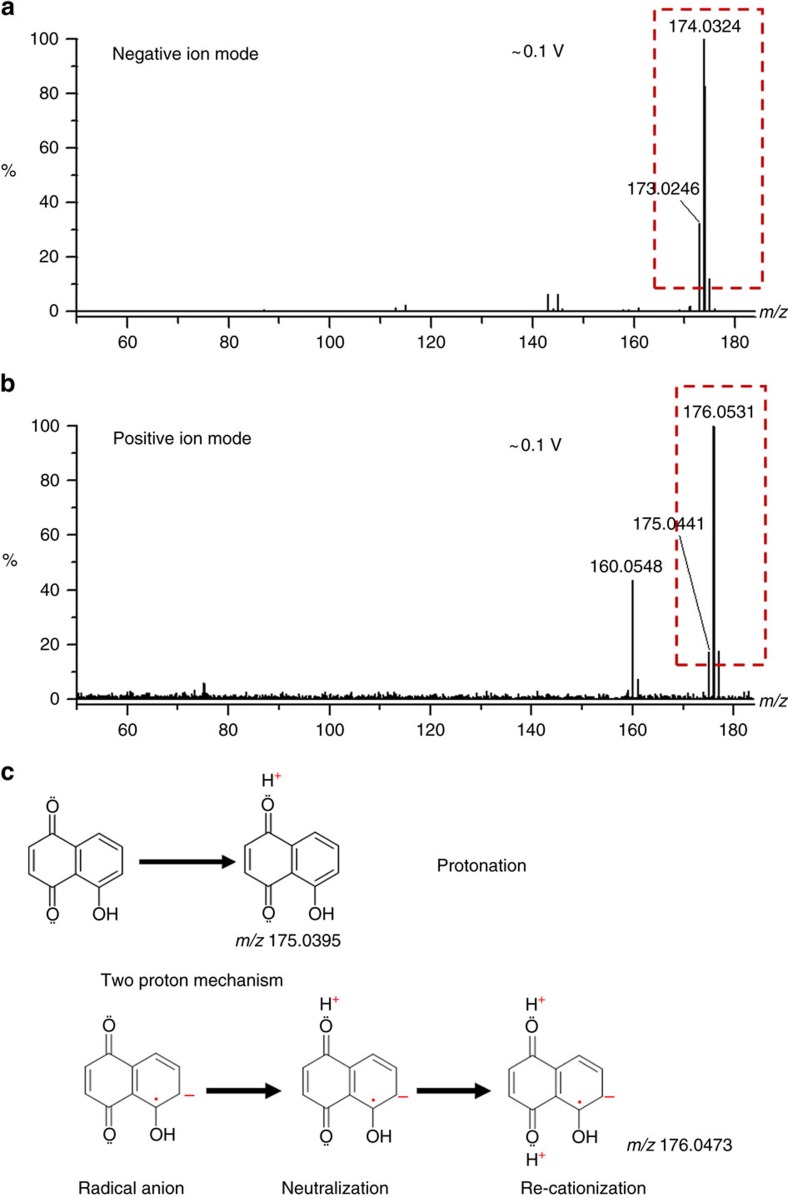
Illustration of associative photoelectron capture on nanoparticles of zinc oxide. (**a**) Mass spectrum of juglone in the negative ion mode. (**b**) Mass spectrum of juglone in the positive ion mode. (**c**) Mechanisms of one-step protonation and two-step protonation. The bias voltage was set as ∼0.1 V for all these experiments.

The formation of an intermediate radical anion was further experimentally confirmed by the dominant molecular ion of juglone at *m/z* 174.0324 Da that was generated on surfaces of zinc oxide nanoparticles. It was observed in the negative ion mode of the mass spectrometer with 0.1 V of bias voltage. Setting of such low bias voltage is aimed to avoid vibrational excitation that may cause break down of ions. The ion at *m/z* 174.0324 Da in Fig. 2a has the same mass as that of the neutral molecule (error: 0.0007 Da). As we know, in the negative ion mode of the mass spectrometer, only negative ions but not neutral molecules can be detected. Observation of the ion with the same mass as that of the neutral molecule unambiguously indicates the capture of photoelectrons. To further validate that a photoelectron was indeed captured by the neutral molecule, the same sample has been analysed in the positive ion mode. As shown in Fig. 2b, an ion at *m/z* 176.0531 Da was observed in addition to usual protonated ion at *m/z* 175.0441 Da. The formation mechanism of these ions was illustrated in Fig. 2c. Compared with the mass of a neutral molecule, the 1.0090 Da mass differences represent the addition of one more proton (error: 0.0012 Da). Because the OH groups of juglone molecules have weak acidity, juglone molecules can generate protons by themselves through de-protonation mechanism with laser irradiation. With two proton mechanism shown in Fig. 2c, the first proton neutralizes a radical anion formed through photoelectron capture and then the second proton causes re-ionization of the neutralized molecule. The protonation process was thought to occur at the oxygen atoms of two carbonyl groups of juglone because of the known basicity of carbonyl groups in organic chemistry^26^. In addition, because negative charges carried on radical anions are delocalized over the ions, the carbon anion does not have basicity and it should not be protonated. However, electrostatic interactions between positive protons and radical anions may facilitate the protonation process. Supplementary Fig. 3 shows that the two proton mechanism was not applicable to baicalein because it has only one carbonyl group. Similar results have been obtained on surfaces of anatase titanium dioxide nanoparticles (Supplementary Fig. 4). These experimental results demonstrate the proton-coupled interfacial photoelectron transfer and the production of radical anions with laser irradiation on semiconductor nanoparticles.

### Dissociative trapping and reactivity of photoelectrons

**Figure 3:**
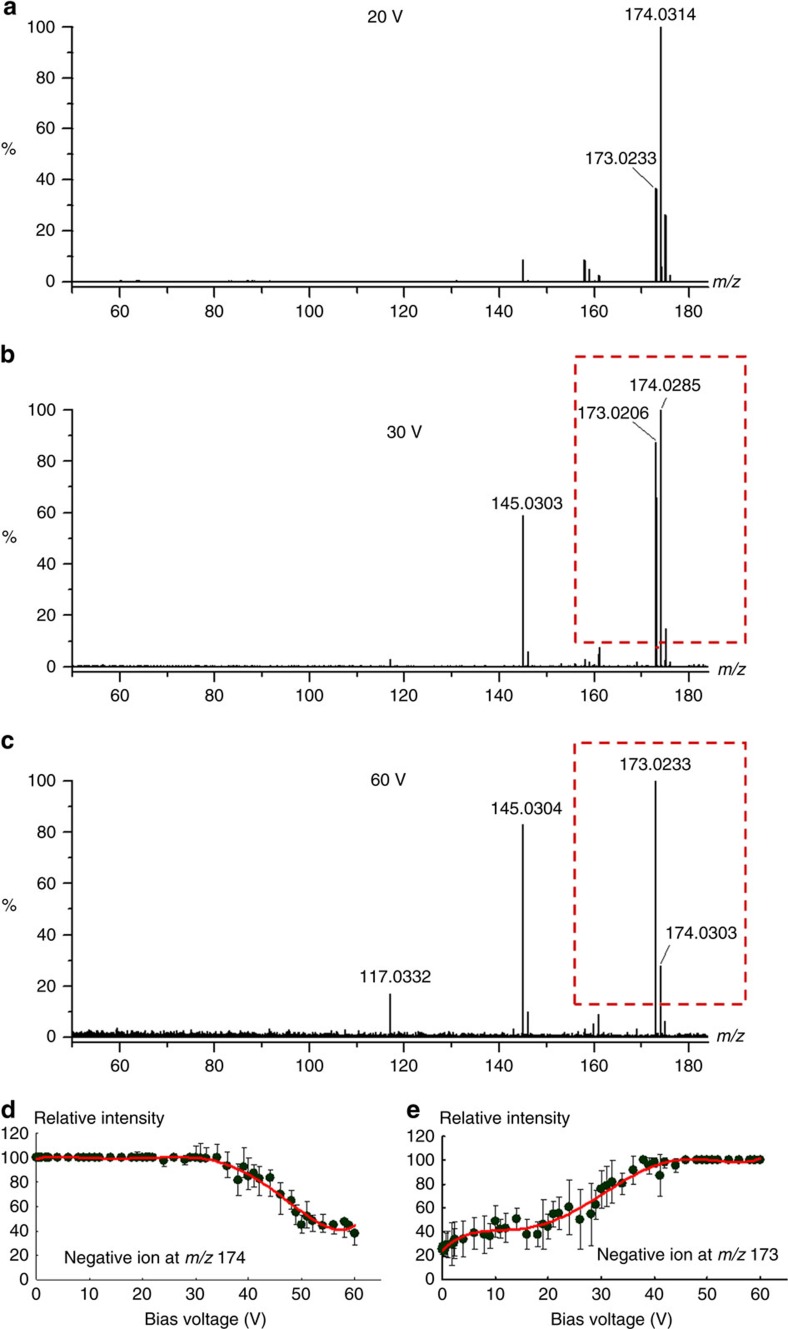
Dissociative photoelectron capture on nanoparticles of zinc oxide under different bias voltages. Mass spectra of juglone were acquired in the negative ion mode under different bias voltages. (**a**) 20 V. (**b**) 30 V. (**c**) 60 V. (**d**) Overall intensity trend of the negative ion at *m/z* 174 Da and (**e**) 173 Da under different bias voltages.

Because of the presence of very active unpaired electrons, intermediate radical anions undergo subsequent chemical bond cleavage and new bond formation. Reactivity of intermediate radical anions is monitored by *in situ* mass spectrometric detection of secondary intermediates or products. When the bias voltage between the sample plate and the aperture increases, kinetic energy of photoelectrons gradually increases which allows sequential cleavage of different bonds with different bond energies. When kinetic energy of photoelectrons is controlled as 20 eV or even lower, the de Broglie wavelength of photoelectrons does not match general bond length of organic molecules. Under such condition, photoelectrons can only be exothermally captured by charge deficient atoms and initiate electron-directed chemical bond cleavage without vibrational activation. Nanoparticles of zinc oxide have been used for experimental demonstration of the reactivity of intermediate radical anions. In Fig. 3a, the radical anion at *m/z* 174.031 Da is dominant at 20 V bias voltage. The ion at *m/z* 173.0233 Da has 1.0081 Da mass shifts than the radical anion at *m/z* 174.0314 Da. The mass differences indicate the loss of an H atom (error: 0.0003 Da). When the bias voltage increases from 0 to 60 V, relative intensities of the ion at *m/z* 174.0314 Da decreases from 100 to ∼20%, while the ion at *m/z* 173.0233 Da increases from ∼20 to 100%, as shown in Fig. 3a–c. Overall intensity trends of these two ions are shown in Fig. 3d and e, respectively. There are three interesting findings from Fig. 3. (1) Loss of an H atom is preferred because of the lowest bond energy and the stability of resultant ions. The ion at *m/z* 173.0233 Da results from the loss of an H atom. It was observed even with only 0.1 V bias voltage because photons of ultraviolet irradiation can provide enough energy for O–H bond cleavage. Delocalization of negative charges over the aromatic ring of resultant ions accounts for the resonance stabilization. (2) Dissociation of radical anions rapidly rises, when the bias voltage was increased to >20 V. Increased bias voltage results in the formation of two ions at 145.0303 Da and 117.0332 Da with 27.9929 Da and 27.9972 Da mass shifts to the radical anion respectively, which represents the losses of CO (error: 0.0020 Da and 0.0023 Da, respectively) through sequential cyclocondensation reactions. The ion at *m/z* 145.0303 Da was observed along with the ion at *m/z* 173.0233 Da when the bias voltage was set as 20 V. When the bias voltage was increased to 30 V, the relative intensity of the ion at *m/z* 145.0303 Da approached ∼70%. The ion at *m/z* 117.0332 Da was not observed until the bias voltage was increased to >60 V. These experimental results indicate that the cyclocondensation reaction needs much more energies than that to break down O–H bonds. (3) When Fig. 3d was compared with Fig. 3e, it was found that there are larger intensity deviations for the ion at *m/z* 173 Da than that of the ion at *m/z* 174 Da, which is in accordance with the internal conversion process demonstrated in Fig. 1e. Because of the internal conversion process, a series of low-lying electronic states with different energies of the radical anion at *m/z* 174 Da results in various degrees of degradation.

**Figure 4:**
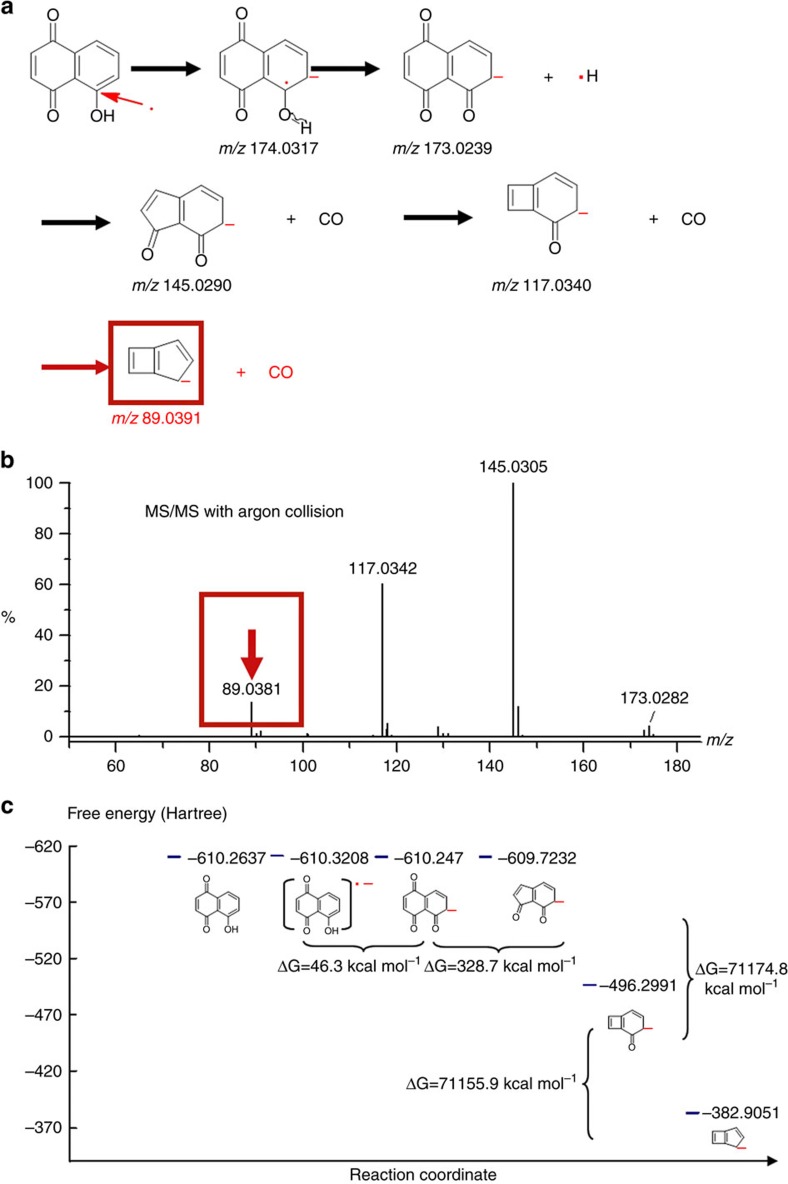
Reactivity of radical anions in dissociative photoelectron capture. (**a**) Degradation pathways to generate negative ions at *m/z* 173.0239, 145.0290, 117.0340 and 89.0391 Da, respectively. (**b**) Tandem MS/MS spectrum of juglone with argon collision activated dissociation. (**c**) The free-energy profile along degradation reaction co-ordinate.

Degradation mechanisms are illustrated in Fig. 4a. It is shown that fragment ions can be generated by either specific α cleavage of O–H bond or sequential losses of CO molecules through cyclocondensation. However, Fig. 3c shows that even with 60 bias voltages, the ion at *m/z* 89.0391 still could not be detected through photoelectron capture dissociation. Instead, as shown in Fig. 4b, it was detected with argon collision activated dissociation. DFT calculation summarized in Fig. 4c indicates that changes in free energies for all these degradation reactions are >0, meaning these degradations cannot proceed spontaneously without additional energies. In fact, there are much larger Δ*G* values for ions at *m/z* 117.0332 Da and 89.0391 Da than that of other ions. It is in accordance with experimentally observed much higher energies needed for those degradations.

It is now clear that capture of photoelectrons results in the formation of stable radical anions over which acquired electrons are delocalized. We have also demonstrated that this mass spectrometric result is in accordance with what we observed in real atmospheric condition. Supplementary Figure 5 shows the pictures for the original juglone solution and the solutions mixed with titanium dioxide nanoparticles under ultraviolet irradiation alone or together with 100 °C heating, respectively. It was found that ultraviolet irradiation alone did not cause the degradation of juglone molecules unless additional energies were provided. To confirm the degradation mechanism shown in Fig. 4a, ^13^C NMR technique has been applied to monitor changes in carbon atoms. Supplementary Figure 6a–c shows that all carbon peaks of carbonyl groups labelled as 1, 2 and 3 were still detected even under ultraviolet irradiation for 5 h. Altogether with ultraviolet irradiation and heating, these peaks disappeared. The disappeared carbon peaks represents the sequential losses of CO molecules. Related ^1^H NMR spectra are shown in Supplementary Fig. 6d–f. This NMR observation is in accordance with what we have shown in the mass spectra of Figs 2 and 3. It also validates the proposed mechanism shown in Fig. 4a. In the mass spectrometer, only when the bias voltage was increased to >20 V, degradation of juglone can be detected. Similar results have also been observed in the solution of juglone suspended with semiconductor nanoparticles of zinc oxide that has been subjected to ultraviolet irradiation in atmospheric condition (Supplementary Fig. 7). With increased energies, intensities of fragment ions with losses of CO molecules increase. In summary, the reactivity of intermediate radical anions is due to unpaired electrons. Stabilization of acquired charges and energies needed for bond cleavage play important roles in unpaired electron initiated reactions.

### Radical initiated polymerization reactions

**Figure 5:**
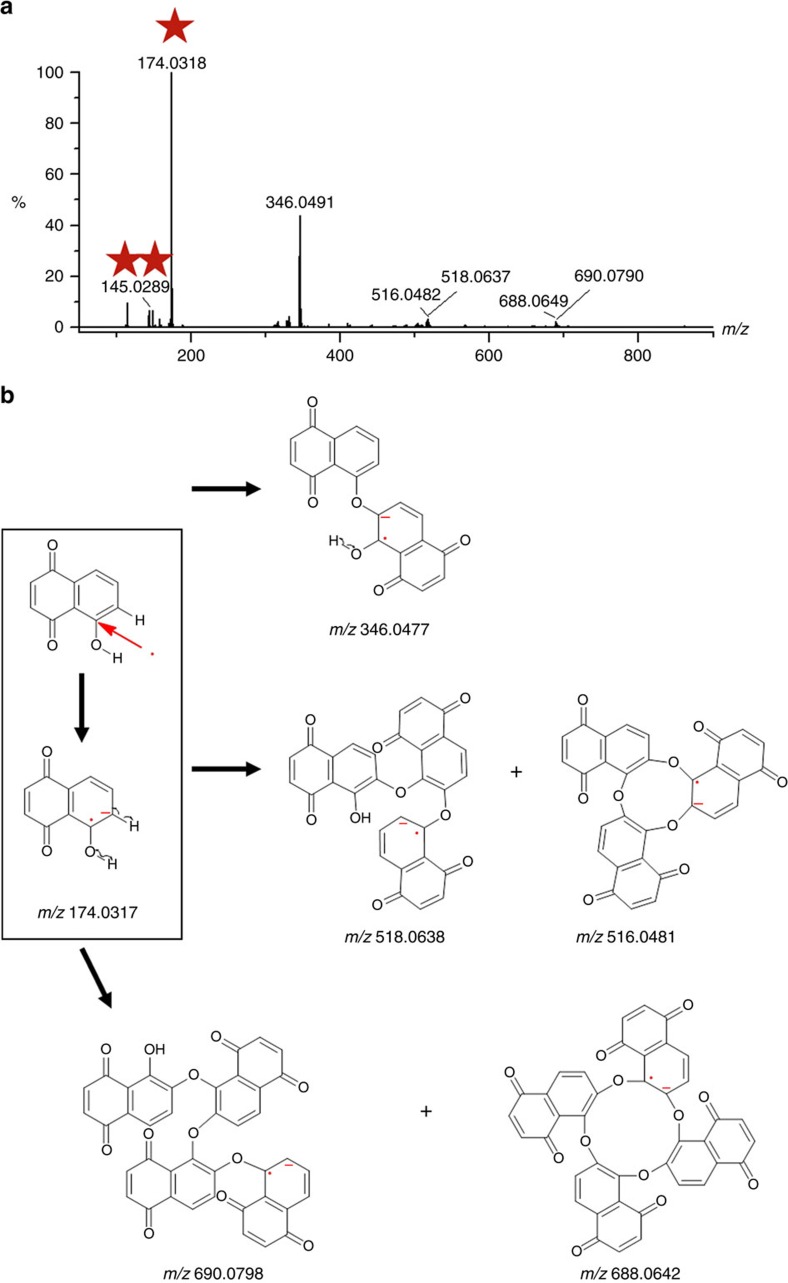
Radical initiated polymerization reactions. (**a**) Mass spectrum of the ions with *m/z* values higher than that of original juglone. (**b**) Pathways for generation of dimmers, trimmers and tetramers of juglone.

In contrast to dissociation, it has been observed that highly reactive radicals can actually also initiate polymerization reactions, which provides another experimental evidence for the occurrence of interfacial photoelectron transfer and the presence of unpaired electrons in resultant species. Figure 5a indicates that there are several ions with *m/z* values much higher that of the radical anion and fragments (labelled as red stars). As shown in Fig. 5b, the highly active unpaired electron present in the radical anion can activate the two α-positioned bonds and cause homolytical cleavage of adjacent C–H and O–H bonds. Then newly formed radicals react with each other to pair electrons and generate stable dimmers (error: 0.0014 Da), trimmers (error: 0.0001 Da) and tetramers (error: 0.0007 Da). Both open-chain structures and ring-shape structures with 2.0155 Da mass shifts have been observed for those trimmers and tetramers. Identities of these polymers are not only validated by their accurate masses but also further confirmed by the ^13^C NMR spectrum (Supplementary Fig. 6). In fact, careful examination of Supplementary Fig. 5 reveals that the colour of the juglone solution became darker under ultraviolet irradiation. In contrast to degradation reactions, this result indicates that new species with larger molecular absorption coefficients have been produced when the juglone solution mixed with titanium dioxide nanoparticles has been subjected to ultraviolet irradiation. Supplementary Figure 8 shows that dimmers, trimmers and tetramers are still present in the solution even with heating.

In particular, it is very interesting that only the open-chain structured dimmer at *m/z* 346.0491 Da was found but the ring-shape structured dimmer theoretically at *m/z* 344.0321 Da was not found. By looking at Fig. 5b, it was recognized that a rigid structure was confined within the aromatic plane. Because of the steric effect, the ring-shape structured dimmer cannot be formed and elongation reactions should be terminated by a hole oxidization mechanism.

### Roles of hydroxyl radicals in degradation pathways

**Figure 6:**
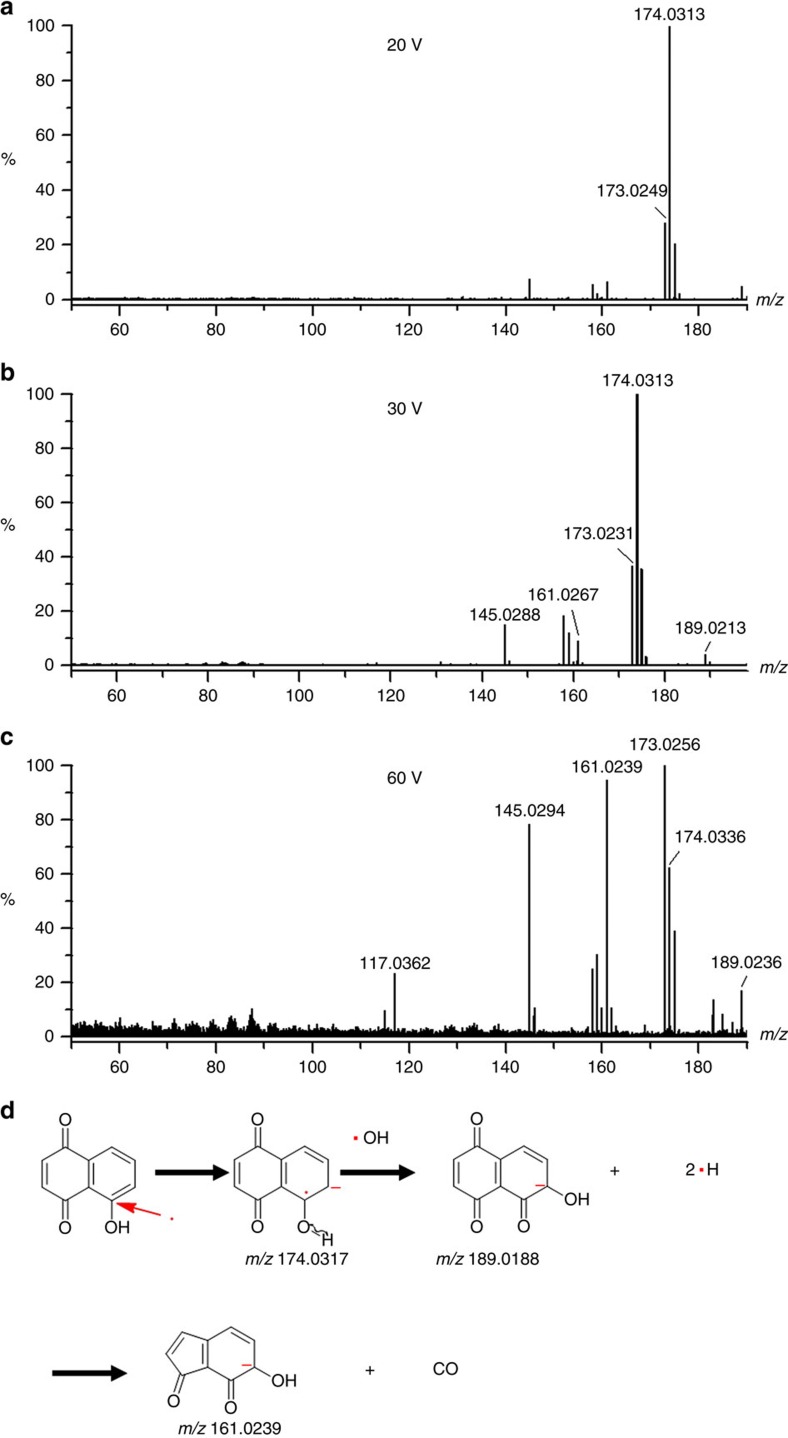
Hydroxyl radical abstraction on nanoparticles of titanium dioxide under different bias voltages. (**a**) 20 V. (**b**) 30 V. (**c**) 60 V. (**d**) Mechanism of the abstraction of a hydrogen atom of C–H bond by hydroxyl radical.

In addition to reductive pathways through either associative or dissociative electron capture and radical initiated polymerization, hole oxidization generated hydroxyl radicals have also been considered as an important intermediate for efficient photo degradation of pollutants^27^,^28^,^29^,^30^,^31^. It has been proposed that hydroxyl radicals can react with other molecules through the abstraction of a hydrogen atom from a C–H bond^32^. However, detection of hydroxyl radicals has been proven difficult because of the rapid degradation of resultant products. In this work, by using the mass spectrometry, intermediate ions are instantly extracted in the static electric field and thus can be detected. Figure 6a–c represents the ions generated on surfaces of titanium dioxide nanoparticles with ultraviolet irradiation at 20 V, 30 V and 60 V bias voltages, respectively. Attention has been attracted to two ions at *m/z* 161.0267 Da and 189.0213 Da. Judged by accurate masses, these two ions are produced by the abstraction of a hydrogen atom from a C–H bond of the ions at *m/z* 145.0290 Da and 173.0239 Da, respectively (error: 0.0028 Da and 0.0025 Da). They have also been observed in the solution of juglone that has been subjected to ultraviolet irradiation in real atmospheric condition (Supplementary Fig. 9), as well as the solution mixed with zinc oxide nanoparticles (Supplementary Fig. 10).

Abstraction of a hydrogen atom from a C–H bond of juglone by a hydroxyl radical is favourable because the lone pair electrons of oxygen atom can be delocalized over the aromatic ring, as shown in Fig. 6d. Resultant hydrogen atom with an unpaired electron is also highly reactive. Mayer *et al*. has reported that the addition of stable 2,4,6-tri-tert-butylphenoxyl radical (tBu_3_ArO·) to air-free toluene solutions (a solvent without acidic proton) of ZnO/e– or TiO_2_/e– (amorphous or anatase) can yield phenol tBu_3_ArOH (ref. 33). A mechanism of proton-coupled electron transfer (PCET) has proposed for explanation of the production of tBu_3_ArOH. Although it has been assumed that active protons likely come from surface hydroxyl groups originated from particle syntheses, the formation of a hydrogen atom from hydroxyl group was not explained. This work provides experimental evidence for the presence of hydroxyl radicals. A hydrogen atom resulting from hydroxyl radical abstraction, instead of a proton, may react with the tBu_3_ArO· radical to produce phenol tBu_3_ArOH. Because *in situ* mass spectrometric detection of gaseous ions was performed in high-vacuum condition, interferences of solvents are eliminated in this work. It has been demonstrated that the abstraction of hydrogen atoms with hydroxyl radicals is independent of the acidity of the juglone solution (Supplementary Fig. 11), which is in accordance with that Mayer *et al*. observed. In fact, hydroxyl group have been widely found on surfaces of different semiconductor nanoparticles such as zinc oxide or titanium dioxide. The presence of –OH has been confirmed with the strong O–H vibrational stretch in the infrared spectra^34^. Hole oxidization of –OH group results in the formation of hydroxyl radicals that can initiate downstream reactions. It has been shown that proton-uncoupled electron transfer process actually is coupled with the hydroxyl group in addition to radical initiated polymerization.

### Intermediates in electron detachment dissociation

**Figure 7:**
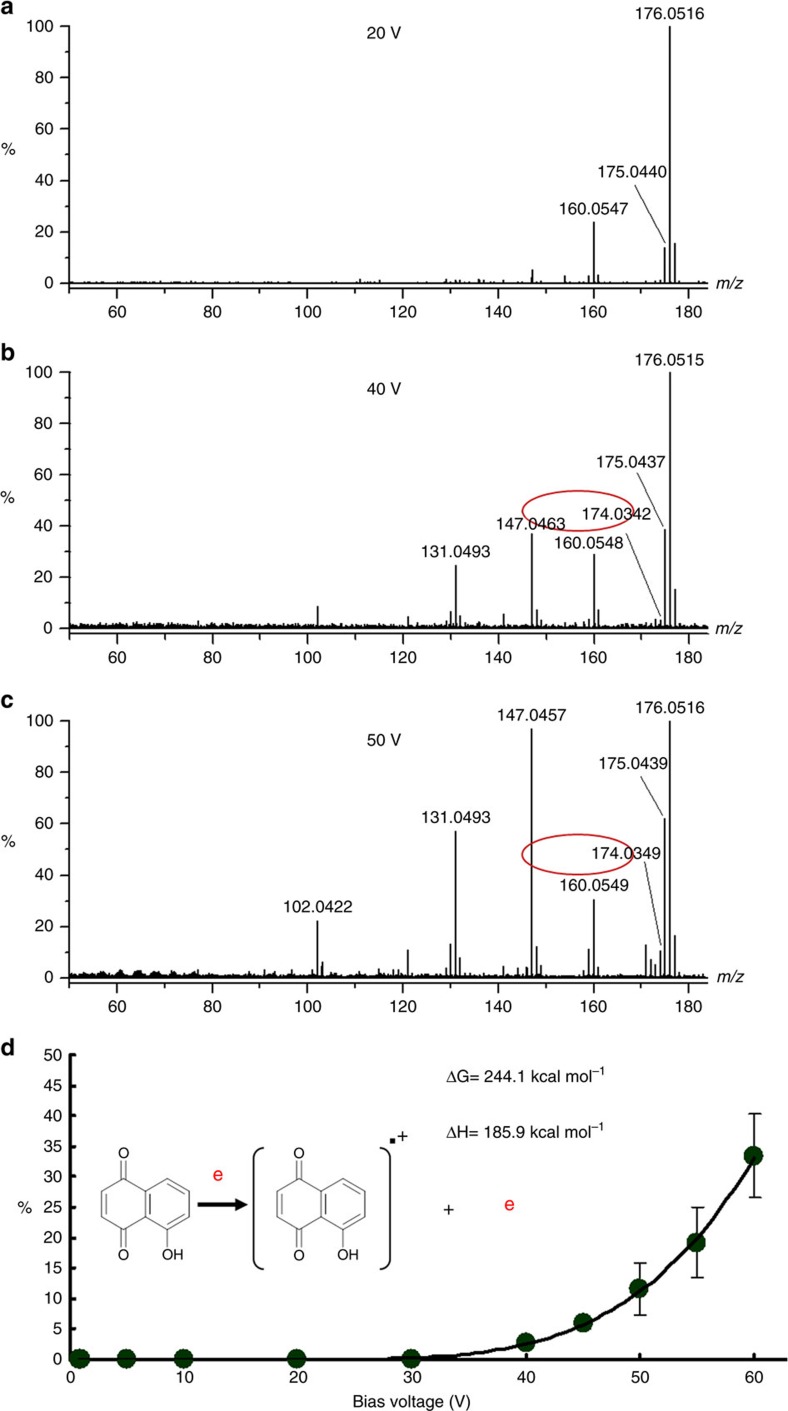
Electron detachment dissociation on nanoparticles of zinc oxide under different bias voltages. (**a**) 20 V. (**b**) 40 V. (**c**) 50 V. (**d**) Overall intensity trend of the ion at *m/z* 174.0317 Da under different bias voltages.

One of the important advantages of mass spectrometry is the versatility to untargetedly detect all ions generated through different mechanisms. When the kinetic energy of photoelectrons was increased to >40 eV by increasing the bias voltage between the sample plate and the aperture, ions resulting from electron detachment dissociation were observed in the positive ion mode of the mass spectrometer. Under low bias voltage such as 20 V, commonly observed ions that are generated through one-step protonation (*m/z* 175.0440 Da) and two-step protonation (*m/z* 176.0516 Da) are shown in Fig. 7a. When the bias voltage approached 40 V, an ion at *m/z* 174.0342 Da indicated with a red oval that has the same mass (error: 0.0025 Da) as that of the neutral molecule of juglone is observed in Fig. 7b. Its intensity increases with increased bias voltage, as shown in Fig. 7c. This ion was formed because of the loss of an electron. DFT calculation shown in Fig. 7d, reveals that ΔG value for electron detachment process is >0. Unlike electron capture dissociation, electron detachment process cannot spontaneously proceed unless additional energy is provided. Comparing exothermal photoelectron capture with endothermal photoelectron impact, ions are generated through different pathways although degradation reactions are also initiated by unpaired electrons. Two protonation of the negative ion at *m/z* 145.0290 Da detected in negative ion mode of the mass spectrometer results in the formation of the ion at *m/z* 147.0446 Da detected in positive ion mode of the mass spectrometer. Occurrence of such proton-coupled electron transfer processes ascribes to the proton affinity of adsorbed molecules and the energy provided by laser irradiation for O–H bond dissociation.

### Imaging of active crystalline facets

**Figure 8:**
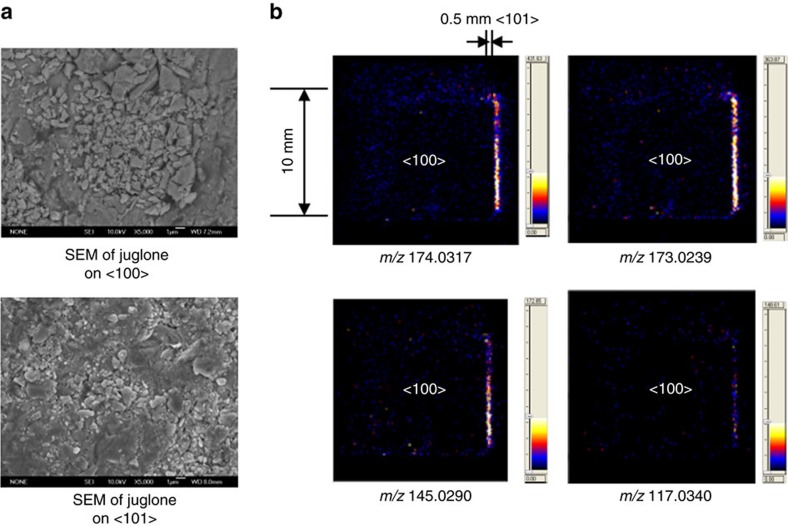
Investigation of crystalline facet-dependent photocatalytic activity of semiconductors. (**a**) Surface characterization of exposed <100> facets and adjacent facets with adsorbed juglone molecules. (**b**) Mass spectrometric imaging of active crystalline facets of rutile titanium dioxide.

Facet-dependent photocatalytic performance of semiconductor nanoparticles has been well recognized recently. Controlled synthesis of photo active single-crystalline semiconductors with desirable exposed facets needs new techniques that can reveal the activity of each crystalline facet. On the basis of the principle of the proposed mass spectrometric approach, active crystalline facets of a single rutile titanium dioxide crystalline have been visualized by scanning the ultraviolet laser across the facet <100> and adjacent facets. In Fig. 8a, scanning electron microscope (s.e.m.) has been used to characterize surfaces of semiconductor crystallines. It was clearly shown that juglone molecules have been adsorbed on both <100> and adjacent facets. However, very weak signals have been observed on the <100> facet. The stronger photocatalytic activity of the adjacent <101> facet is proven by the detection of much stronger ions of juglone at *m/z* 174.0317 Da, 173.0239 Da, 145.0290 Da and 117.0340 Da in Fig. 8b. Among these ions, the one at *m/z* 117.0340 Da has the lowest intensity because of higher energy needed to produce this ion. In addition, observation of the strong original peak without degradation at *m/z* 174.0317 provides further experimental evidence on the formation of stable anionic ground states through ultrafast internal conversion as that has been reported. By using first-principles calculations, the surface energies of rutile <100> facet and <101> facet have been reported as 0.67 J m^−2^ and 1.01 J m^−2^, respectively with PAW10 pseudopotential (Projector Augument Wave)^35^. The much higher surface energy of <101> face is in accordance with the experimentally observed higher photocatalytic activity on <101> facet. However, it should be indicated that the crystal-facet dependency of activities is also affected by surface defects, as well as energy levels of the conduction bands.

To further demonstrate that photocatalytic activity is not only associated with surface properties, but also highly dependent on properties of adsorbed molecules, organochlorine 4, 4′-DDT has been used as an example. As shown in Supplementary Fig. 12, in contrast to juglone, the original peak of 4, 4′-DDT without degradation at *m/z* 351.9147 Da has much lower intensity than that of the degradation product at *m/z* 280.9692 Da on the <101> facet. It indicates that capture of the photoelectron by 4, 4′-DDT rapidly initiates downstream chemical bond cleavage. Because of the electron attracting effect of chlorine atoms, radical anions produced by photoelectron capture of 4, 4′-DDT is not as stable as that of juglone.

## Discussion

The mass spectrometry-based approach is capable for monitoring of interfacial photoelectron transfer and imaging of active crystalline of semiconductors. In the mass spectrometer, recombination of electron–hole pairs is prevented by the built-in static electric field and interferences of atmospheric N_2_ and O_2_ are eliminated in the intrinsic high vacuum condition. Once photoelectrons are captured by adsorbed electron receptors, resultant radical anions are pulled out of surfaces and detected by the mass spectrometer. Because kinetic energies of photoelectrons are dependent on this electric field, reactivity of radical anions can be evaluated with secondary intermediates and degradation products that are generated with increased kinetic energies of photoelectrons. Unpaired electron-directed bond cleavage specifically at α position has been demonstrated as the major degradation pathway. Energies needed for bond cleavage and stabilization of resultant charges play important roles in the formation and detectability of various intermediates and products. By using the presented mass spectrometric approach, proton-coupled electron transfer and proton-uncoupled electron transfer with radical initiated polymerization as well as hydroxyl radical abstraction have been observed. They have been found in both associative and dissociative photoelectron capture pathways. In the case of hydroxyl radical abstraction, it is the hydrogen atom instead of a proton that is coupled with the interfacial electron transfer. When high energy is provided, degradations occur through electron detachment dissociation pathway as well. In the positive ion mode of the mass spectrometer, proton-coupled electron transfer has been observed because of the proton affinity of adsorbed molecules and photo ionization of O–H bond. Imaging of active crystalline facets has been achieved by scanning ultraviolet laser across different facets and reconstruction of mass spectra. The activity of each facet can be monitored by original ions, intermediates and degradation product ions. It has been shown that mass spectrometric approach should be very useful in materials sciences in addition to biological sciences.

## Methods

### Reagents and apparatus

LC–MS grade water and isopropanol were purchased from Fisher Scientific (Bridgewater, NJ, USA). Nanoparticles of anatase titanium dioxide, zinc oxide (<100 nm BET or <50 nm XRD), rutile titanium dioxide single crystal substrate with exposed <100> facet (10 × 10 × 0.5 mm) and juglone were purchased from Sigma-Aldrich (MO, USA). 4, 4′-dichlorodiphenyltrichloroethane was purchased from Dr Ehrenstorfer GmbH (Bgm, Schlosser, Germany). Acetone and ethanol was purchased from Guoyao (Tianjing, China). In the low-mass region of negative ion mode, the mass spectrometer was calibrated with free fatty acids. Conductive sticky alumina tape was purchased from Junke (Shanghai, China). All standard fatty acids (including C4:0, C6:0, C8:0, C10:0, C12:0, C14:0, C16:0, C18:0, C20:0 and C22:0) were purchased from Nu-ChekPrep Inc (MN, USA). In the low-mass region of positive ion mode, the mass spectrometer was calibrated with polyethylene glycol (PEG) standard that was purchased from Waters (Milford, USA). Surfaces of rutile titanium dioxide crystalline were characterized with a Jeol JSM-6700F scanning electron microscope (MA, USA). NMR experiments were performed on a Varian 600 MHz NMR (CA, USA).

### Mass spectrometric analysis

Mass spectrometric experiments were performed on a Waters MALDI Synapt G2 HDMS system (Milford, USA) mass spectrometer. Data have been acquired in resolution mode (resolution is around 40,000) and mass accuracy is 0.3 p.p.m. To ensure high-mass accuracy during the whole data acquisition process, the mixture solution containing free fatty acids or PEG was used as the lock mass for internal calibration in negative ion mode and positive ion mode, respectively. Bias voltages between the sample plate and aperture are adjustable between 0 and 100 V. Voltages on the sample plate and the aperture were set as 87 V and 107 V respectively for routine analysis. Nanoparticles were suspended in isopropanol solution (10 mg ml^−1^), and then spotted in the sample wells of the sample plate. After nanoparticles were air dried, juglone dissolved in a solution of acetone (2 mg ml^−1^) was spotted onto the surfaces of nanoparticles. When the laser beam shots on surfaces of nanoparticles, photo generated radical anions, intermediates and degradation products of photocatalytic reactions are simultaneously extracted into the vacuum and detected by a time-of-flight mass analyser. For imaging analysis, the semiconductor crystalline was soaked in the acetone solution of juglone (2 mg ml^−1^), and dried in air before put into the instrument. For *in vitro* experiments, juglone was dissolved in a solution containing 50% water and 50% acetone (100 mg ml^−1^). Nanoparticles of titanium dioxide or zinc oxide were suspended in this solution (10 mg ml^−1^) that was subjected to ultraviolet irradiation (365 nm) for 50 min. Then the suspension was centrifuged at 16,000*g*, and the supernatant was analysed.

Laser pulses of the mass spectrometer were derived from an Nd: YAG high-repetition laser head (355 nm) that is equipped in the source chamber of the mass spectrometer. Laser pulse energy, pulse width and fire rate are 100 μJ, 200 Hz^−1^, 3 ns and 200 Hz, respectively. Laser influx is adjustable between 0 and 500 units. For routine analysis, laser influx was set as 200 units. Laser beam size is adjustable from 5 to 250 μm. In this work, the laser beam size was fixed at ∼15 μm, corresponding to average power densities of 7.5 × 10^−20^ J μm^−2^. Mass spectrometry imaging was achieved through scanning the laser beam across <100> facets and adjacent facets. The step size has been set as 80 × 80 μm. For each pixel, the acquisition time is 1 s. Potential differences between the sample plate and the aperture were set as 20 V for all imaging experiments. HDI software (Waters, US) was used for imaging analysis.

### Computational methodology

To investigate the dynamics of photoelectrons, DFT was used as a quantum mechanistic modelling method to study the electronic structures of molecules. Ground state electronic energies were calculated by using Gaussian software (Wallingford, CT, USA)^36^. Structures were first optimized with B3LYP/6-31G^+^ (d) basis set. Ground state electronic energies were calculated at the B3LYP/6-31G^+^ (d) level. Charge distribution of molecules was evaluated with natural bond orbital calculation.

### Data availability

The authors declare that the data supporting the finding of this study are available within the article and its Supplementary Information files. For NMR and high-resolution mass spectrometry analysis of the compounds in this article, see Supplementary Fig. 6; Figs 2, 3, 4, 5, 6, 7. These data can be obtained free of charge from the web of this journal. Other data related to this study are also available from the corresponding author on reasonable request.

## Additional information

**How to cite this article:** Zhong, H. *et al*. Mass spectrometric monitoring of interfacial photoelectron transfer and imaging of active crystalline facets of semiconductors. *Nat. Commun.* **8,** 14524 doi: 10.1038/ncomms14524 (2017).

**Publisher's note:** Springer Nature remains neutral with regard to jurisdictional claims in published maps and institutional affiliations.

## Supplementary Material

Supplementary Information Supplementary Figures
