# Supplementary material for: Mass spectrometric monitoring of interfacial photoelectron transfer and imaging of active crystalline facets of semiconductors
Source: Nat Commun. 2017 Feb 22;8:14524. doi: 10.1038/ncomms14524 (PMC5322523; doi:10.1038/ncomms14524)
Supplement: Supplementary Information — Supplementary Figures [file ncomms14524-s1.pdf]

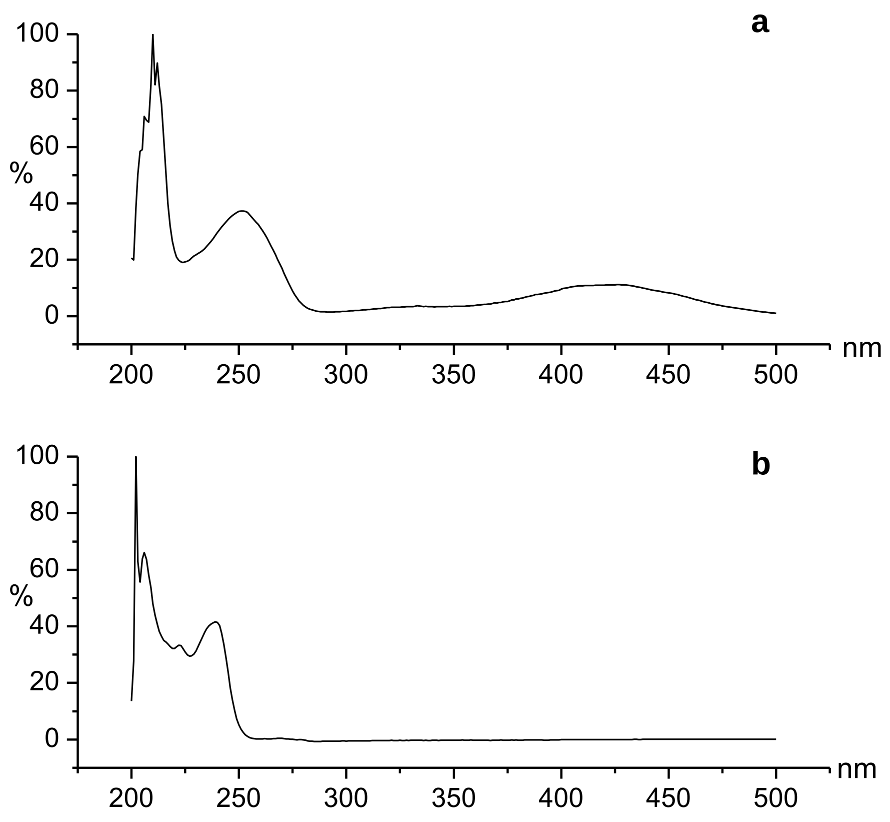

Supplementary Figure 1. UV absorption spectra in the solution of ethanol (1.25 μg mL^{-1}). (a) juglone. (b) DDT.

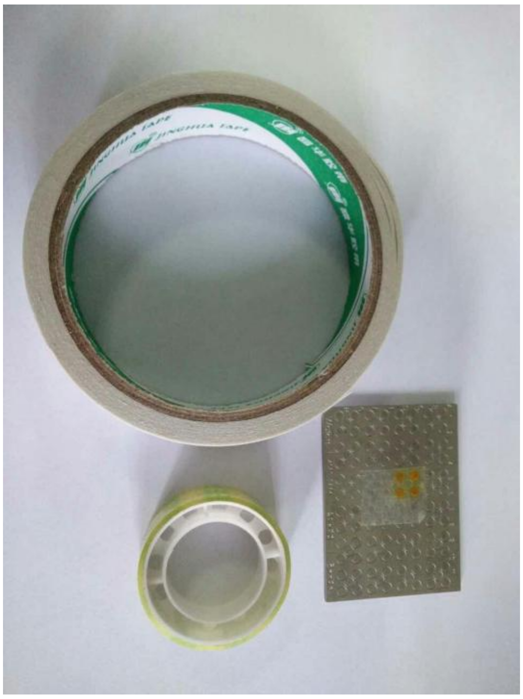

Supplementary Figure 2. The picture of juglone that was deposited on insulated glue tapes.

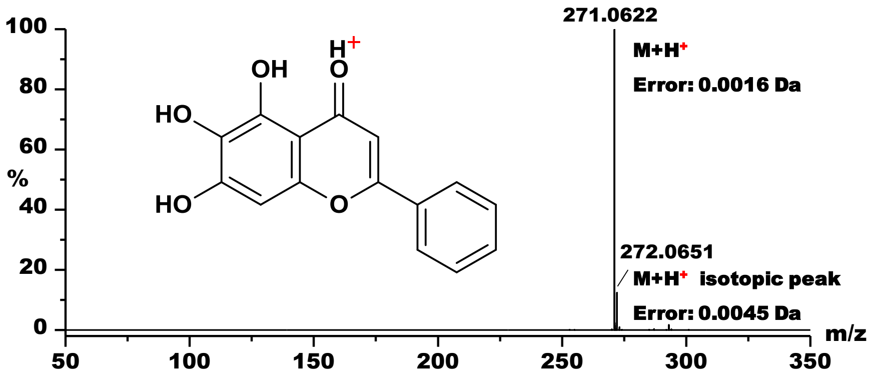

Supplementary Figure 3. Mass spectra of baicalein adsorbed on surfaces of zinc oxide nanoparticles with ultraviolet irradiation in positive ion mode. Two protonated ions were not observed.

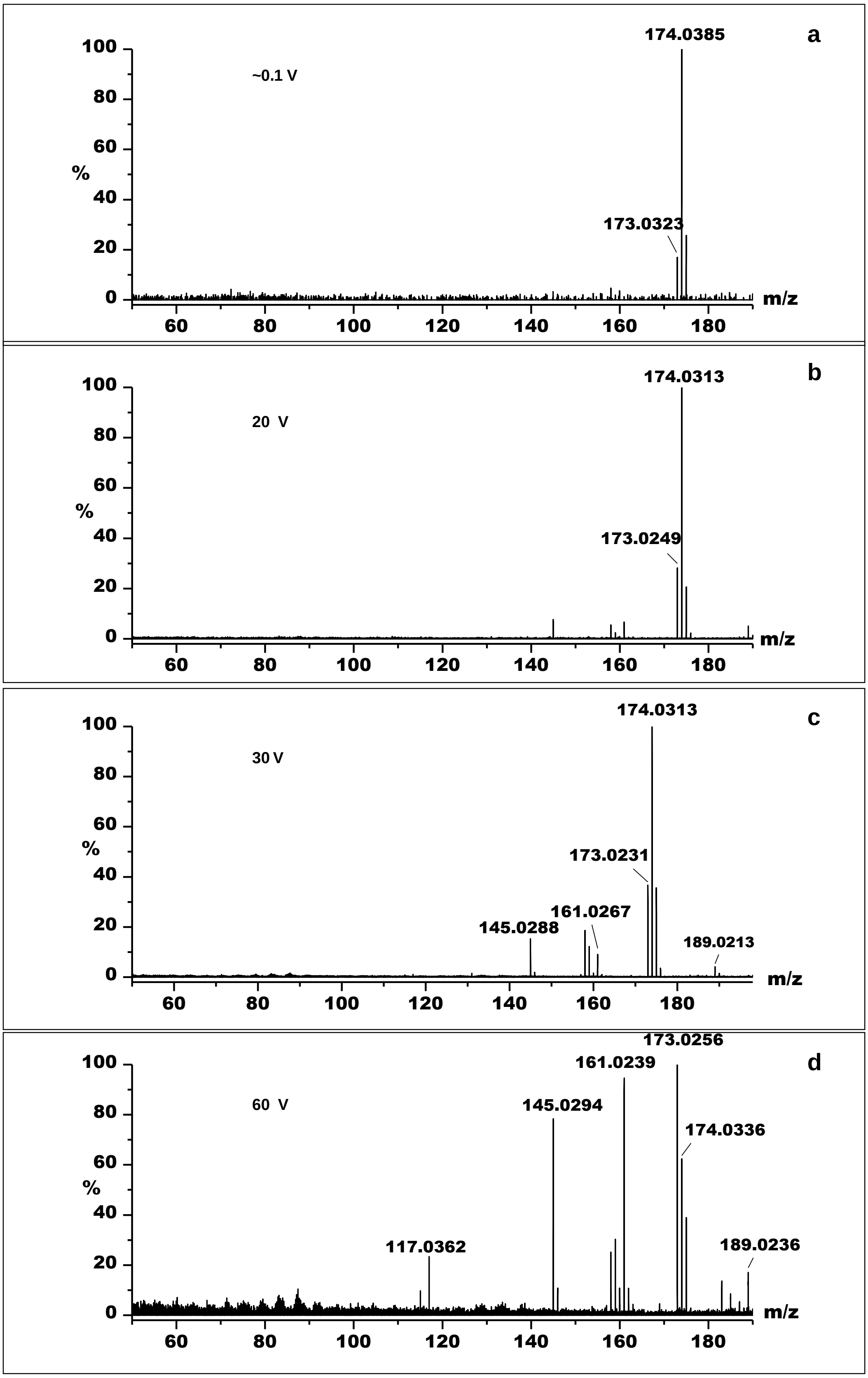

Supplementary Figure 4. Mass spectra of juglone adsorbed on surfaces of anatase titanium dioxide nanoparticles under ultraviolet irradiation with different bias voltages. (a) ~ 0.1 V. (b) 20 V. (c) 30 V. (d) 60 V.

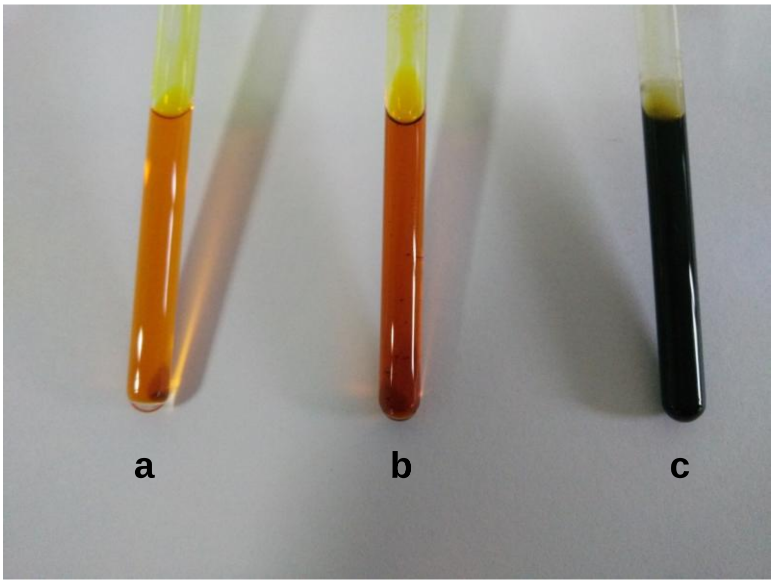

Supplementary Figure 5. Pictures juglone solutions. (a) original juglone solution. (b) juglone solution that has been mixed with anatase titanium dioxide nanoparticles under ultraviolet irradiation for 5 hours alone or (c) along with heating to 100 ° C for 1 hour in atmospheric condition.

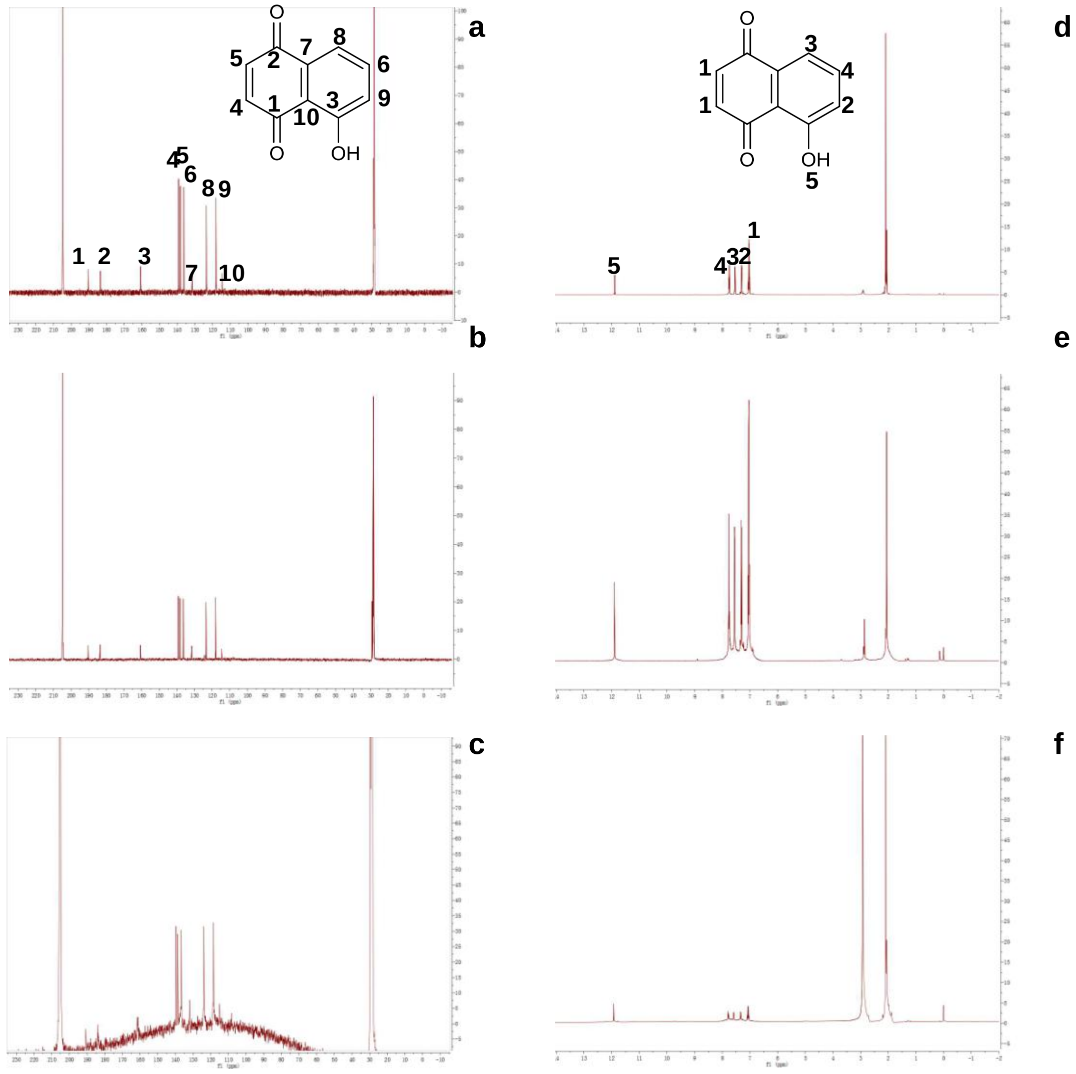

Supplementary Figure 6. Characterization of the compounds with NMR. (a)^{13}C NMR spectra of original juglone solution and juglone solution that has been mixed with anatase titanium dioxide nanoparticles under ultraviolet irradiation for 5 hours alone (b) or along with heating to 100° C for 1 hour in atmospheric condition (c). (d-f) ^{1}H NMR spectra of the same compounds as that of ^{13}C NMR. Acetone-D6 has been used as the solvent for all NMR experiments.

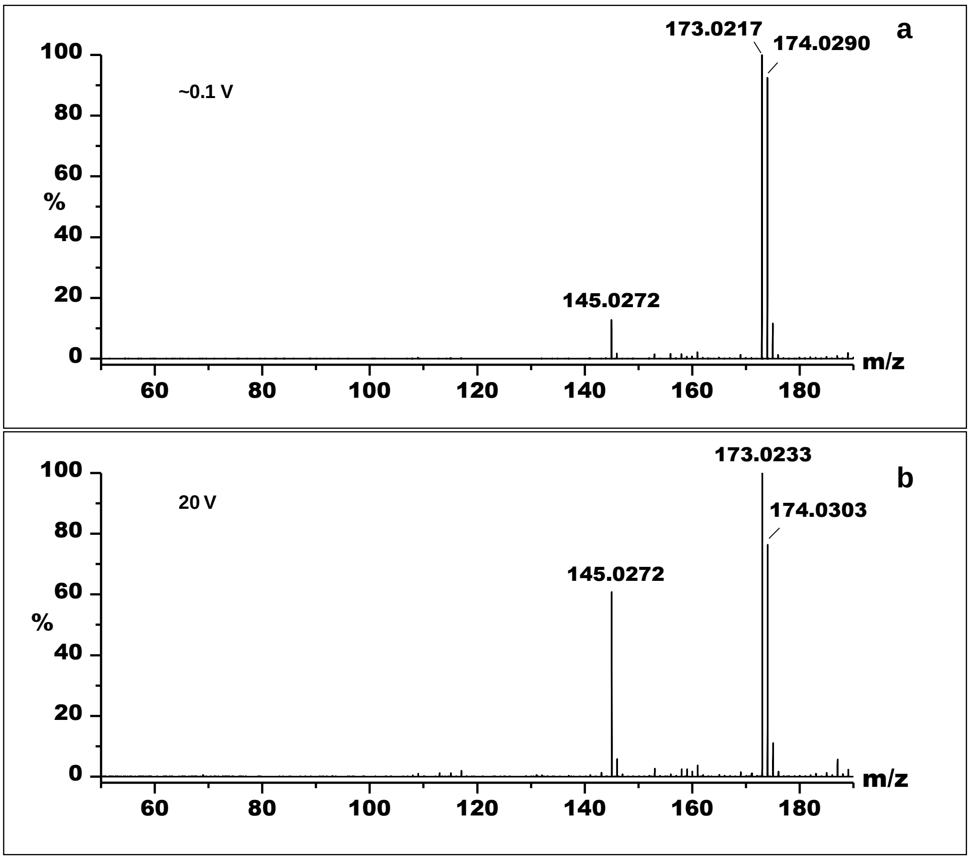

Supplementary Figure 7. Mass spectra of juglone adsorbed on surfaces of zinc oxide nanoparticles under ultraviolet irradiation for 5 hours in atmospheric condition with different bias voltages. (a) ~ 0.1 V. (b) 20 V.

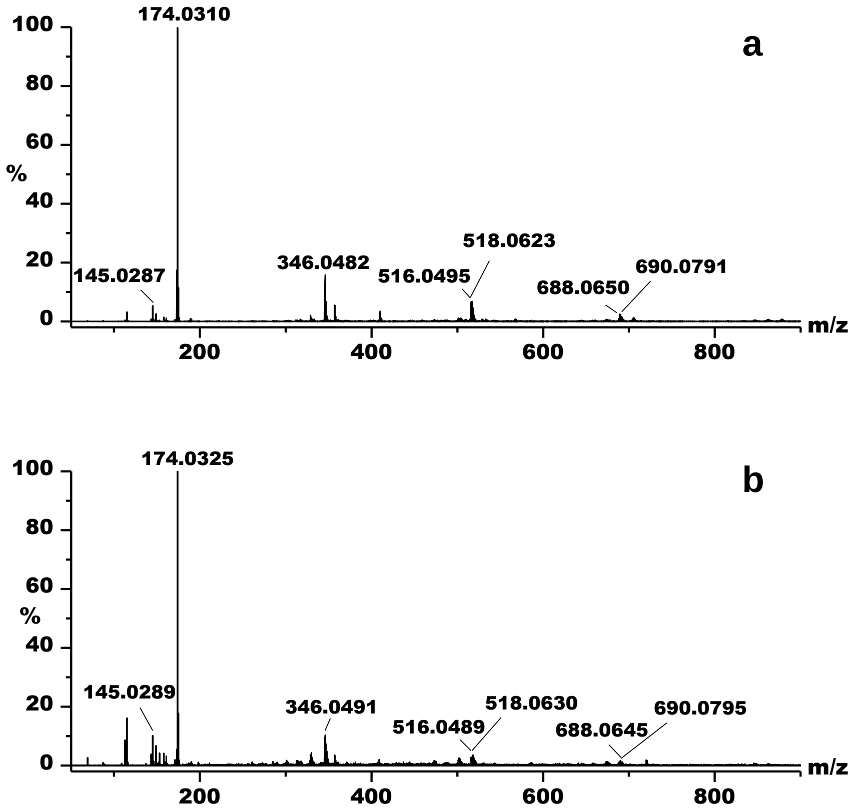

Supplementary Figure 8. Mass spectra of juglone solutions that have been mixed with titanium dioxide nanoparticles and subjected to ultraviolet irradiation alone for 5 hours (a) or together with 100 ° C heating for 1 hour (b) in atmospheric condition. The bias voltages between the sample plate and the aperture of the mass spectrometer is 20 volts.

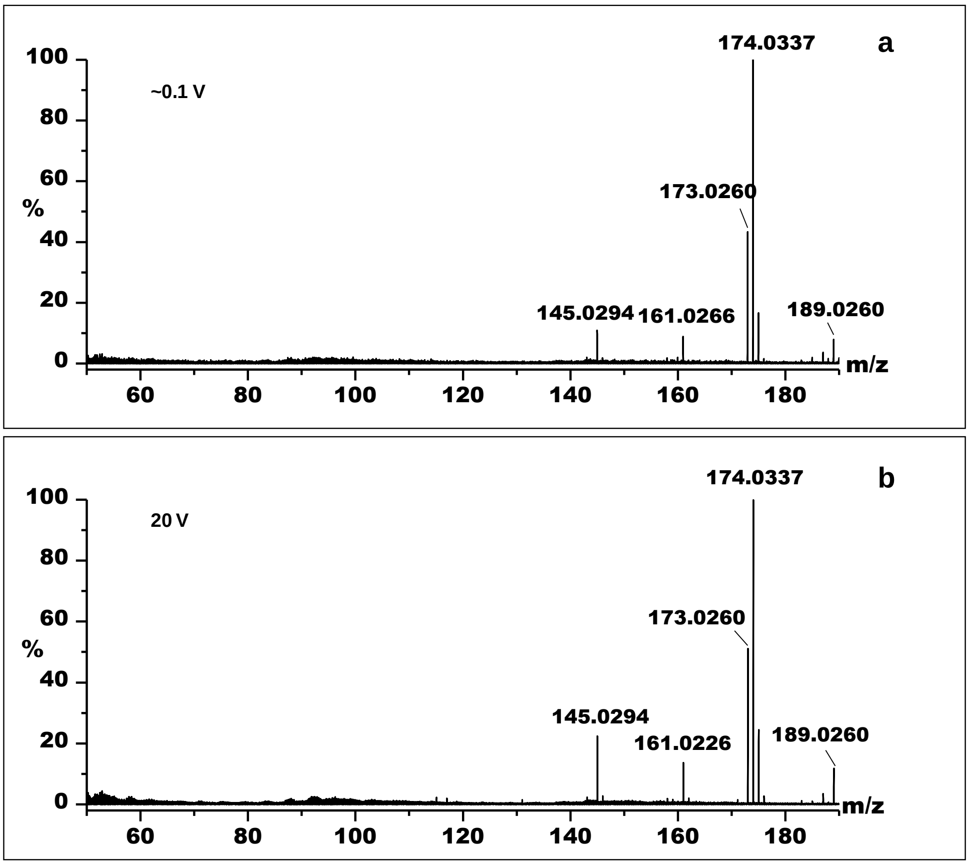

Supplementary Figure 9. Zoomed mass spectra of juglone adsorbed on surfaces of anatase titanium dioxide nanoparticles under ultraviolet irradiation in atmospheric condition with different bias voltages. (a) ~ 0.1 V. (b) 20 V.

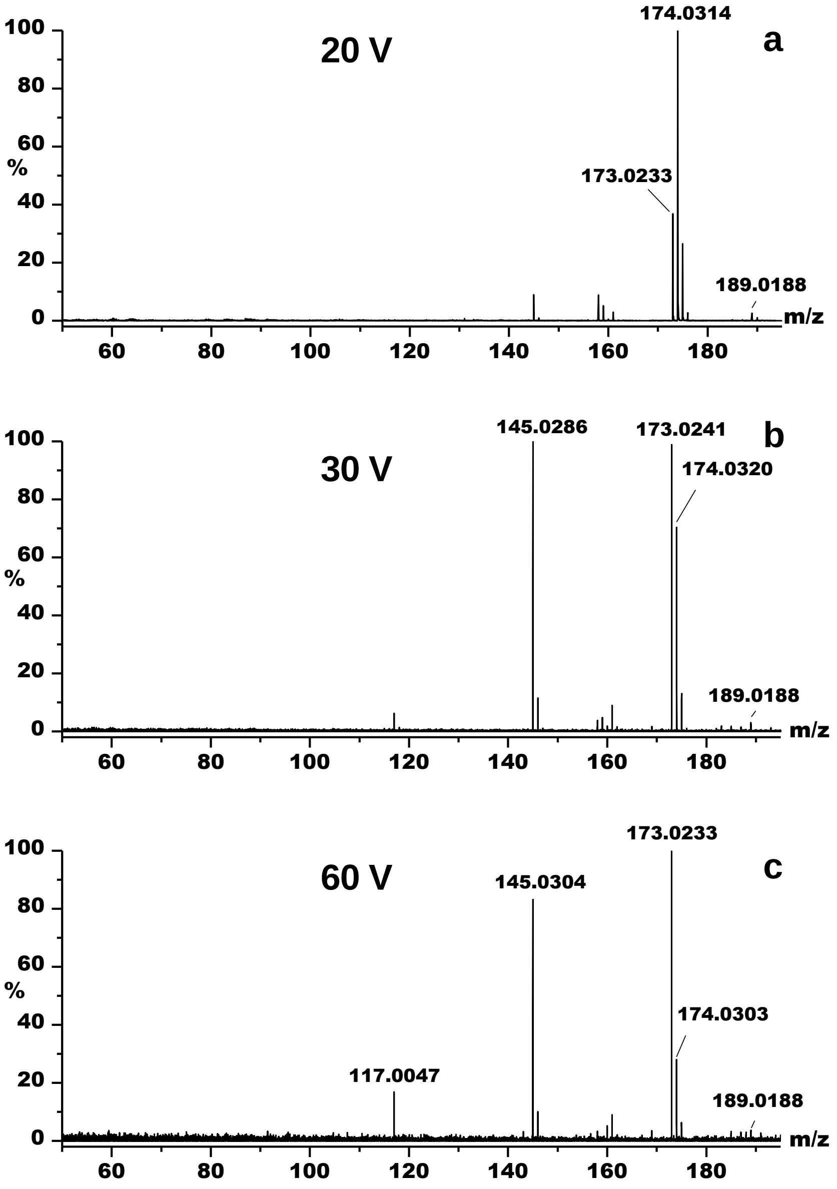

Supplementary Figure 10. Mass spectra of juglone adsorbed on surfaces of zinc oxide nanoparticles at different bias voltages. (a) 20 volts. (b) 30 volts. (c) 60 volts.

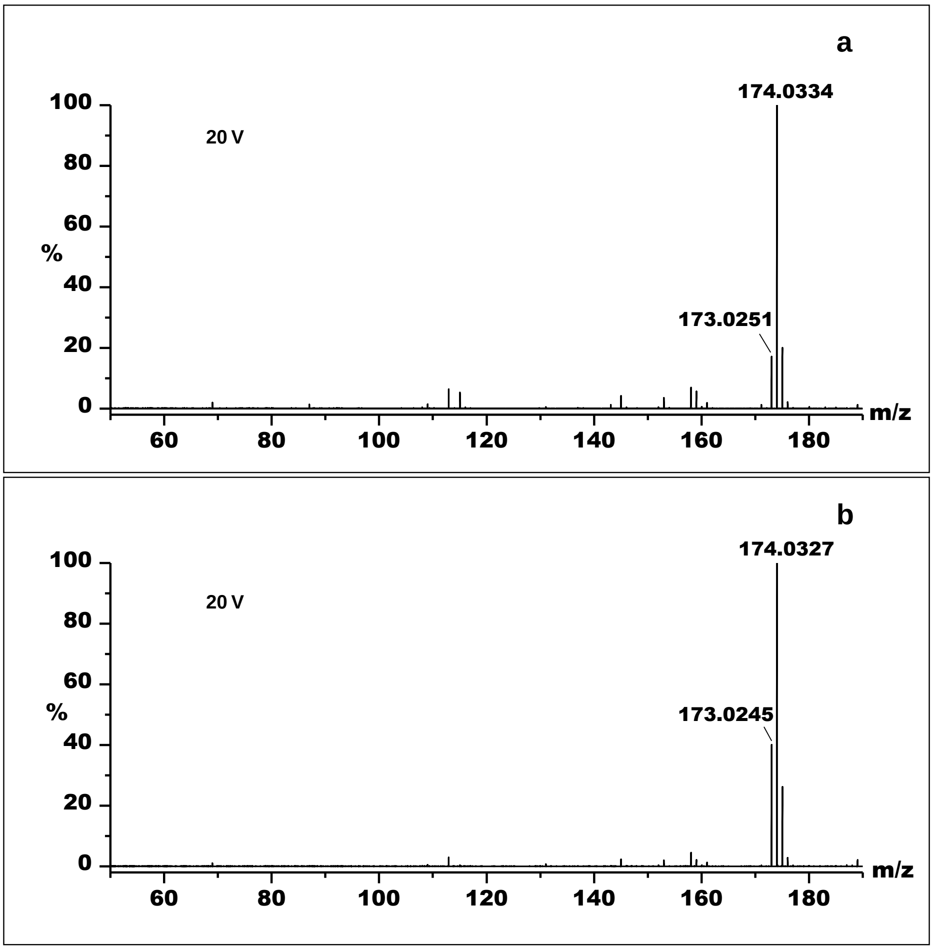

Supplementary Figure 11. Mass spectra of juglone adsorbed on surfaces of zinc oxide nanoparticles. The solution of juglone contains 0.1% acetic acid (a) or 1% ammonia (b).

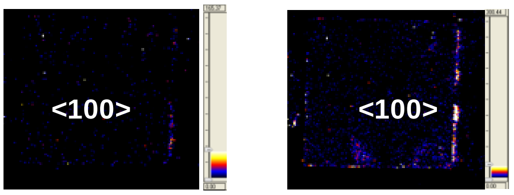

4, 4'-DDT **Degradation products**

Supplementary Figure 12. Mass spectrometric imaging of DDT and degradation products on exposed rutile <100> facets.
